# Adherence to National Standard Treatment Guidelines in the management of hypertension and associated factors among healthcare providers at public district hospitals in Dar es Salaam, Tanzania

**DOI:** 10.1371/journal.pgph.0005818

**Published:** 2026-07-09

**Authors:** Gasper Singfrid Mung’ong’o, Daniel Msilanga, Paul Alikado Sabuni, Humphrey Godwin Medarakini, Gladys Reuben Mahiti, Rose Mpembeni

**Affiliations:** 1 Department of Development Studies, Muhimbili University of Health and Allied Sciences, Dar es Salaam, Tanzania; 2 Department of Internal Medicine, Muhimbili University of Health and Allied Sciences, Dar es Salaam, Tanzania; 3 Department of Epidemiology and Biostatistics, Catholic University of Health and Allied Sciences, Mwanza, Tanzania; 4 Department of Emergency Medicine, Muhimbili University of Health and Allied Sciences, Dar es Salaam, Tanzania; 5 Department of Epidemiology and Biostatistics, Muhimbili University of Health and Allied Sciences, Dar es Salaam, Tanzania; PLOS: Public Library of Science, UNITED STATES OF AMERICA

## Abstract

Hypertension affects 15–25% of adults in Tanzania, yet fewer than 30% of treated patients achieve blood pressure control. Poor control contributes to stroke, heart failure, and renal disease. The National Standard Treatment Guidelines (NSTGs), 6th edition (2021), provide guidance for management, but healthcare provider adherence to the guidelines remains unclear. A mixed-methods cross-sectional study was conducted at five public district hospitals in Dar es Salaam, Tanzania between 1^st^ April and 31^st^ May 2025. Quantitative data from patient records assessed healthcare provider adherence to pharmacological treatment, complication monitoring, and comorbidity screening, categorized as complete, partial, or non-adherent. Modified Poisson regression identified associated patient and provider factors. Qualitative data from 11 in-depth interviews were thematically analysed to explore barriers and facilitators to adherence. The median age of the 397 patients was 55 years (IQR: 52–61), 79.1% were female, 40.0% had at least one comorbidity, and 65.2% were overweight. Only 26.2% were managed with complete adherence to NSTGs. Higher adherence was associated with follow-up visits (aPR = 5.81; 95% CI: 1.59–21.27), Grade 1 hypertension (aPR = 3.87; 95% CI: 1.99–7.53), Grade 2 hypertension (aPR = 5.06; 95% CI: 1.52–16.73), comorbidities (aPR = 2.35; 95% CI: 1.25–4.41), specialist cadre (aPR = 7.00; 95% CI: 1.05–46.55), and provider experience of 5–10 years (aPR = 3.25; 95% CI: 1.17–9.03) and >10 years (aPR = 3.91; 95% CI: 1.20–12.73). Barriers included outdated guidelines, limited resources, staffing shortages, and inadequate training, while facilitators included mentorship, peer collaboration, accessible guidelines, and continuing medical education. Healthcare provider adherence to NSTGs in management of hypertension was low. Strengthening guideline implementation through updated recommendations, provider training, improved resource availability, and supportive supervision may enhance adherence and improve patient outcomes.

## Introduction

Hypertension remains a major global public health challenge and one of the leading causes of mortality and disability worldwide. The number of people living with hypertension has doubled from an estimated 650 million in 1990 to 1.3 billion by 2019 [1,2]. Despite the global efforts, hypertension control rates remain alarmingly low, with approximately 23% of individuals on treatment achieving adequate blood pressure control [2].

In Tanzania, the burden of NCDs has grown steadily over the past decades. NCDs accounted for 19% of total DALYs in 1990, rising to 34% by 2015, and currently contribute to nearly one-third of all deaths, with a substantial proportion occurring prematurely between ages 30 and 70 [3,4]. Furthermore, among patients on treatment less than 30% attain target blood pressure levels, placing them at substantial risk for life-threatening complications and premature mortality [4,5,6,7].

Recognizing these trends, Tanzania’s National Strategic Plan for the Prevention and Control of Non-Communicable Diseases (2021–2026) identifies hypertension control as a national priority and aligns with both the Health Sector Strategic Plan V and the Universal Health Coverage agenda. A key strategy within this framework is the standardization of care given to patients with Hypertension [8].

The National Standard Treatment Guidelines (NSTGs) 6^th^ edition developed by the Ministry of Health in 2021, provide evidence-based guidance for the diagnosis and management of hypertension in Tanzania [9,10]. However, the extent of healthcare provider adherence to the NSTGs in the management of hypertension remains unclear. Therefore, this study aimed to evaluate adherence to the NSTGs among healthcare providers in public district hospitals in Dar es Salaam. The study also aimed to identify patient- and provider-related factors associated with adherence, as well as the barriers and facilitators influencing healthcare providers’ adherence to the NSTGs in management of Hypertension.

## Materials and methods

### Study design

A cross-sectional study design employing a convergent parallel mixed-methods approach. The quantitative component assessed the level of healthcare provider adherence to the NSTGs and identified statistically associated provider- and patient-related factors, while the qualitative component explored the barriers and facilitators influencing this adherence. The findings from both components were analyzed separately and integrated during interpretation.

### Study area

The study was carried out at five public district hospitals in Dar Es Salaam region one from each district. These are: Kivule district hospital (Ilala), Ubungo district hospital (Ubungo), Mbagala rangi tatu hospital (Temeke), Kigamboni district hospital (Kigamboni) and Mabwepande district hospital (Kinondoni). These hospitals are located in Dar Es Salaam, an urban setting where there is a growing population of patients with Hypertension and hence an appropriate setting to evaluate healthcare provider adherence to the NSTGs in management of these patients [11].

### Study population

The study population included patients diagnosed with hypertension who attended outpatient clinics at the study facilities between 1 January and 31 December 2024, and healthcare providers of all cadres involved in prescribing and managing hypertension. Quantitative data were extracted from patient records, while qualitative data were collected through in-depth interviews with healthcare providers. Both quantitative data extraction and qualitative interviews were conducted at the study facilities between 1 April and 31 May 2025.

### Inclusion and exclusion criteria

1. Inclusion criteria
  i. Patients with Hypertension who were attended from January 2024 to December 2024 at the selected public district hospitals and have case file.
  ii. Healthcare providers in the public district hospitals who take part in the management of patients with hypertension and agreed to be part of the study
2. Exclusion criteria
  i. Patients with case files that do not have a documented diagnosis and management provided between January 2024 and December 2024.
  ii. Healthcare providers with less than one year of experience working in the selected hospitals as they have limited exposure to the Tanzanian health system and the NSTGs.

### Sample size and sampling procedures

For the quantitative analysis, all eligible records of patients with hypertension from January to December 2024 were included using a census approach to maximize statistical power and minimize selection bias; therefore, no prior minimum sample size was calculated. Duplicate entries were identified using unique Medical Record Numbers and removed to ensure that each patient contributed only once to the dataset.

For the qualitative component, eleven healthcare providers participated in in-depth interviews. Sampling followed the principle of data saturation. After the ninth interview, no new themes emerged, and two additional interviews were conducted to confirm thematic stability. By the eleventh interview, redundancy was reached, indicating sufficient depth and breadth of data across provider cadres and facility contexts.

Patient data were extracted from the Government of Tanzania Hospital Management Information System (GOTHOMIS) outpatient department registers. Providers were selected purposively based on their direct involvement in hypertension management at either Non-Communicable Disease (NCD) clinics or general outpatient departments. Only those with at least one year of service and who gave written informed consent to participate were included.

### Study variables

The dependent variable was healthcare provider adherence to NSTGs in hypertension management, measured through a structured checklist applied to retrospective patient records covering the three months preceding the most recent clinic visit.

Adherence was assessed in three domains:

1. **Pharmacological management:** Consistency of prescribed antihypertensive medications with NSTG recommendations based on patient classification, comorbidities, and crisis status.
2. **Monitoring for complications:** Performance of recommended investigations such as ECG, echocardiography, renal function tests, urinalysis, fundoscopy, and relevant laboratory tests.
3. **Screening for comorbidities:** Screening for diabetes mellitus, dyslipidemia, and obesity through glucose tests, lipid profiles, and BMI assessment.

Each item was scored dichotomously (1 = adherent, 0 = non-adherent). The adherence score was computed as a percentage of total possible points. Independent variables included patient characteristics (age, sex, BMI, payment mode, visit type, hypertension grade, comorbidities) and provider characteristics (age, sex, professional cadre, years of experience, and facility).

The qualitative component explored barriers and facilitators influencing provider adherence to NSTGs.

#### Quantitative data collection.

Data were collected by the principal investigator using a structured digital checklist developed on the Open Data Kit (ODK) a free, open-source mobile data collection platform. Eligible records for patients with Hypertension were identified via GOTHOMIS registers and verified through NCD clinic and laboratory registers. The checklist captured patient demographics, clinical characteristics, investigations, and treatment in line with NSTG recommendations. Data were entered in real time into the Open Data Kit (ODK) platform and securely uploaded to a central server for analysis.

#### Qualitative data collection.

In-depth interviews were conducted in Swahili by the principal investigator using a structured guide. All interviews were audio-recorded and supplemented with detailed field notes capturing non-verbal cues and contextual observations. Recordings were transcribed verbatim and translated into English by trained bilingual research assistants.

### Data management

Audio recordings were stored securely on password-protected Google Drive folders accessible only to the investigator. Transcripts were anonymized before translation. Quantitative data extracted from Open Data Kit (ODK) were exported to Microsoft Excel and stored in encrypted drives before analysis in Stata version 17. Regular backups were maintained to ensure data security and integrity.

### Validity and reliability

The quantitative data collection tool was developed based on the 6th Edition NSTGs and reviewed by two clinical experts (a nephrologist and an emergency physician) for content validity. The tool was pretested on 50 files of patients with Hypertension at a private outpatient clinic to assess clarity and usability, leading to minor revisions. Face validity was confirmed by three independent medical practitioners who reviewed the final checklist for relevance and comprehensiveness.

### Trustworthiness

Trustworthiness was ensured through the application of four key criteria. Credibility was enhanced by prolonged engagement of principal investigator in the field, peer debriefing, and triangulation with observational data to ensure accurate representation of participants’ views. Transferability was supported through rich, detailed descriptions of the study context and participant selection, enabling readers to assess the applicability of the findings to other settings. Dependability was maintained by establishing an audit trail that documented all methodological steps, ensuring consistency and replicability of the research process. Finally, confirmability was achieved through peer debriefing and member checking, which helped minimize researcher bias and ensure that the findings accurately reflected participants’ perspectives.

### Data analysis

Data were analyzed using Stata version 17. Descriptive statistics summarized participant characteristics: means and standard deviations for normally distributed variables, medians and interquartile ranges for skewed data, and frequencies and proportions for categorical variables.

Adherence scores were calculated using:


$$\begin{array}{c} \text{Adherence score}=\;(\text{Points scored}/\text{Total points available})\times \text{1}\text{0}\text{0}\% \end{array}$$

Scores were categorized as complete (≥65%), partial (50–64.9%), or non-adherent (<50%), consistent with prior regional studies that assessed composite provider adherence to hypertension guidelines using similarly structured multi-domain checklists in comparable sub-Saharan African settings [12–14].

Bivariate analyses were conducted to identify variables significantly associated with adherence (p < 0.05). These variables, along with age and sex, were included in the multivariable model. Statistical significance was defined as p < 0.05.

### Qualitative data analysis

Thematic analysis was conducted using ATLAS.ti (version 22) - a qualitative data analysis software. Transcripts were read repeatedly to ensure familiarity with the data. Relevant statements were coded inductively, grouped into subthemes, and then aggregated into major themes representing barriers and facilitators to adherence. Coding and theme development were iterative, with continuous comparison to ensure internal coherence and alignment with study objectives.

### Ethical issues

Ethical approval was obtained from the Muhimbili University of Health and Allied Sciences (MUHAS) Research and Publications Committee (approval number DA.282/298/01.C/2694) on 11 March 2025. Administrative permission was granted by the Regional Medical Officer, District Medical Officers, and facility leadership. Written informed consent was obtained from all healthcare providers who participated in the in-depth interviews.

During data collection, the research team accessed patient medical records containing identifiable information. However, no personal identifiers were recorded or retained. Patient data were anonymized at the point of extraction, and individuals were identified only using unique medical record numbers for study purposes. Confidentiality was ensured through anonymization of data and secure data handling procedures.

## Results

### Patient characteristics

**Fig 1:**
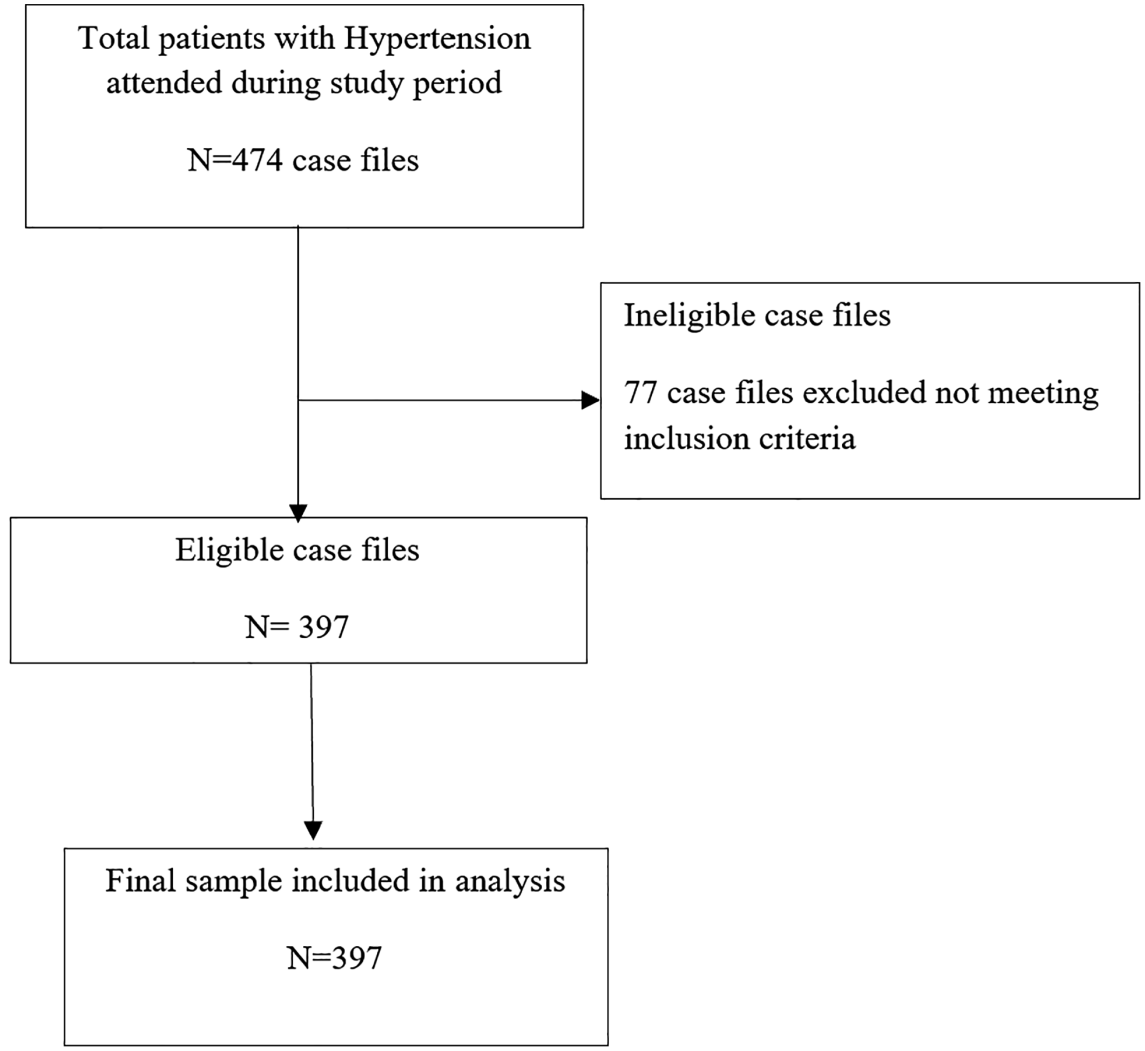
Enrollment of case files in the study.

A total of 474 case files with unique medical record numbers of patients with Hypertension attended at the public district hospitals from January 2024 to December 2024 were obtained from the GOTHOMIS OPD registers. Of these, 397 case files (83.8%) contained complete information required for inclusion. The enrollment process is reflected in Fig 1.

The median age of patients was 55 years (IQR: 52–61), with the majority (233; 58.7%) aged 45–60 years, and 39 patients (9.8%) aged below 45 years. Most patients were female (314; 79.1%). The mean body mass index (BMI) was 27.34 kg/m² (SD: 3.08), with 259 (65.2%) overweight and 81 (20.4%) obese. Regarding payment mode, 265 patients (66.8%) paid out-of-pocket, while 71 (17.9%) were covered by national health insurance. Follow-up visits accounted for 353 (88.9%) of encounters. Blood pressure control was achieved in 166 patients (41.8%), while 21 (5.3%) presented in hypertensive crisis. The median number of antihypertensive drugs used was 2 (range 1–4), with 268 patients (67.5%) using more than one drug. Comorbidities were present in 159 patients (40.0%), with obesity (20.4%) and diabetes mellitus (15.1%) being most common. These are displayed on Table 1.

**Table 1:** Demographic and clinical characteristics of the patients (n = 397).

Variable	Number	Percentage
**Age (median, IQR)**	55, 52-61	
**Age group**		
Less than 45	39	9.8%
45-60	232	58.4%
Above 60	126	31.7%
**Sex**		
Female	314	79.1%
Male	83	20.9%
**Body mass index (mean, SD)**	27.34, 3.083	
**Body mass index category**		
Normal	57	14.4%
Overweight	259	65.2%
Obese	81	20.4%
**Payment mode**		
National health insurance	71	17.9%
Government exception	24	6.1%
iCHF	37	9.3%
Cash	265	66.8%
**Type of the visit**		
New case	44	11.1%
Follow up visit	353	88.9%
**Grade of hypertension**		
Controlled	166	41.8%
Grade 1	188	47.4%
Grade 2	22	5.5%
Grade 3	21	5.3%
**Hypertensive crises**		
Not in crisis	376	94.7%
Hypertension emergency	14	3.5%
Hypertension urgency	7	1.8%
**Number of antihypertensives (median, range)**	2, 1-4	
**Number of antihypertensives**		
One	129	32.5%
More than one	268	67.5%
**Comorbidity condition**		
No	238	60.0%
Yes	159	40.0%
**Types of comorbidity condition Ϯ (n = 397)**		
Diabetes	60	15.1%
Heart failure	35	8.8%
Pregnancy induced	17	4.3%
Asthma	21	5.3%
Arrhythmias	9	2.3%
Dyslipidemias	26	6.5%
Obesity	81	20.4%

Hints: Ϯ = multiple response.

### Healthcare provider characteristics

A total of 26 healthcare providers who attended patients with hypertension included in the study were recruited from five district hospitals (Table 2). Kigamboni, Kivule, and Mabwepande each contributed four providers, while Mbagala Rangi Tatu and Ubungo each contributed seven. The mean age of the providers was 36.25 years (SD = 8.45), with the majority aged 21–30 years (n = 12). Females (n = 15) outnumbered males. The most common professional cadre was medical doctor (n = 16), while specialists were few (n = 3). The mean duration of work experience was 6.69 years (SD = 3.61), with most providers having less than five years of experience (n = 12). These are displayed on Table 2.

**Table 2:** Demographic characteristics of the healthcare providers (n = 26).

Variable	Number of healthcare providers
**Facility name**	
Kigamboni district hospital	4
Kivule district hospital	4
Mabwepande district hospital	4
Mbagala rangi tatu hospital	7
Ubungo district hospital	7
**Age (mean, SD)**	36.25, 8.449
**Age group**	
21-30	12
31-40	8
Above 40	6
**Sex**	
Male	11
Female	15
**Cadre**	
Clinical officer	4
Assistant medical officers	3
Medical doctor	16
Specialist	3
**Working experience (mean years, SD)**	6.69, 3.610
Less than 5 years	12
5–10 years	10
More than 10 years	4

For the qualitative part of the study, In depth interviews were conducted with 11 healthcare providers: 4 clinical officers, 2 assistant medical officers (AMOs), 4 medical doctors (MDs), and one specialist (physician).

**Fig 2:**
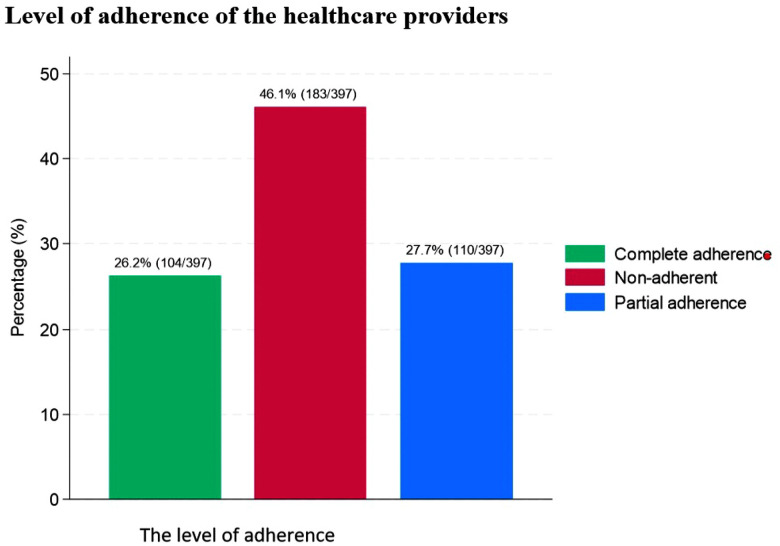
Healthcare provider adherence to NSTGs in management of Hypertension.

Fig 2 shows the level of adherence of healthcare providers to the guidelines in management of Hypertension.

### Level of adherence of the healthcare providers

#### Patient factors associated with adherence of healthcare providers.

Modified Poisson regression revealed that follow-up visits were significantly associated with guideline-concordant management compared to new cases (aPR = 5.81; 95% CI: 1.59–21.27; p = 0.008). Higher grades of hypertension were also positively associated with adherence, including Grade 1 (aPR = 3.87; 95% CI: 1.99–7.53, p < 0.001) and Grade 2 (aPR = 5.06; 95% CI: 1.52–16.73, p = 0.008). Presence of comorbidities was significantly associated with a higher prevalence of guideline-concordant management (aPR = 2.35; 95% CI: 1.25–4.41; p = 0.008).

Table 3 shows the patient factors and their association to healthcare provider adherence to the guidelines.

**Table 3:** Patient factors associated with adherence of healthcare providers to the NSTGs in the management of hypertension.

Variable	Total	Adherence status		Unadjusted PR δ		Adjusted PR δ	
		No n (%)	Yes n (%)	PR, 95% CI	p-value	PR, 95% CI	p-value
**Age group**							
Less than 45	39	32 (82.1)	7 (18.0)	1		1	
45-60	232	171 (73.7)	61 (26.3)	1.37, 0.53–3.53	0.515	0.84, 0.27–2.61	0.766
Above 60	126	90 (71.4)	36 (28.6)	1.60, 0.59–4.29	0.348	0.59, 0.17–2.03	0.406
**Sex**							
Female	314	231 (73.6)	83 (26.4)	1		1	
Male	83	62 (74.7)	21 (25.3)	0.98, 0.53–1.81	0.952	1.47, 0.71–3.08	0.302
**Body mass index category**							
Normal	57	40 (70.2)	17 (29.8)	1			
Overweight	259	201 (77.6)	58 (22.4)	0.68, 0.33–1.39	0.289		
Obese	81	52 (64.2)	29 (35.8)	126, 0.55–2.88	0.577		
**Payment mode**							
Cash	265	204 (77.0)	61 (23.0)	1			
Insurance	132	89 (67.4)	43 (32.6)	1.31, 0.77–2.23	0.315		
**Type of the visit**							
New case	44	40 (90.9)	4 (9.1)	1		1	
Follow up visit	353	253 (71.7)	100 (28.3)	3.23, 1.07–9.78	**0.038**	5.81, 1.59–21.27	**0.008**
**Grade of hypertension**							
Controlled	166	139 (83.7)	27 (16.3)	1		1	
Grade 1	188	131 (69.7)	57 (30.3)	3.21, 1.77–5.84	**<0.001**	3.87, 1.99–7.53	**<0.001**
Grade 2	22	12 (54.6)	10 (45.5)	5.27, 1.81–15.33	**0.002**	5.06, 1.52–16.73	**0.008**
Grade 3	21	11 (52.4)	10 (47.6)	3.88, 1.33–11.30	**0.013**	2.78, 0.76–10.13	0.122
**Comorbidity condition**							
No	238	194 (81.5)	44 (18.5)	1		1	
Yes	159	99 (62.3)	60 (37.7)	2.98, 1.75–5.08	**<0.001**	2.35, 1.25–4.41	**0.008**

Hints: PR = Prevalence Ratio; 95% CI = 95 percent confidence interval; δ = Adjusted by cluster, i.e., healthcare providers; sigma_u = 0.993; rho = 0.23, chi = 30.95 (p < 0.001).

### Healthcare providers’ factors associated with adherence

Provider cadre and experience influenced adherence. Specialists demonstrated significantly higher adherence compared to clinical officers (aPR = 7.00; 95% CI: 1.05–46.55; p = 0.004). Providers with 5–10 years of experience (aPR = 3.25; 95% CI: 1.17–9.03; p = 0.024) and more than 10 years of experience (aPR = 3.91; 95% CI: 1.20–12.73; p = 0.023) were also more likely to adhere. Age and sex showed no significant association after adjustment. Table 4 presents provider-related factors associated with adherence.

**Table 4:** Healthcare providers’ factors associated with adherence of healthcare providers to the NSTGs in the management of hypertension (n = 397).

Variable	Total patient attended	Adherence status		Unadjusted PR δ		Adjusted PR δ	
		No n (%)	Yes n (%)	PR, 95% CI	p-value	PR, 95% CI	p-value
**Age group**							
21-30	140	120 (85.7)	20 (14.3)	1		1	
31-40	158	100 (63.3)	58 (36.7)	2.92, 1.08–7.86	**0.034**	0.48, 0.15–1.52	0.214
Above 40	99	73 (73.7)	26 (26.3)	2.21, 0.76–6.51	0.147	0.86, 0.30–2.47	0.783
**Sex**							
Male	160	117 (73.1)	43 (26.9)	1		1	
Female	237	176 (74.3)	61 (25.7)	0.79, 0.29–2.07	0.624	1.28, 0.70–2.31	0.422
**Cadre**							
Clinical officer	23	21 (91.3)	2 (8.7)	1		1	
Assistant medical officers	39	34 (87.2)	5 (12.8)	1.56, 0.25–9.76	0.637	0.64, 0.09–4.27	0.645
Medical doctor	237	191 (80.6)	46 (19.4)	2.45, 0.51–11.69	0.262	1.48, 0.31–7.04	0.624
Specialist	98	47 (48.0)	51 (52.0)	11.66, 2.31–58.99	**0.002**	7.00,1.05–46.55	**0.004**
**Working experience (mean years, SD)**							
Less than 5 years	144	128 (88.9)	16 (11.1)	1		1	
5–10 years	177	117 (66.1)	60 (33.9)	3.45, 1.41–8.37	**0.006**	3.25, 1.17–9.03	**0.024**
More than 10 years	76	48 (63.2)	28 (36.8)	4.70, 1.61–13.73	**0.005**	3.91, 1.20–12.73	**0.023**

Hints: PR = Prevalence Ratio; 95% CI = 95 percent confidence interval; δ = Adjusted by cluster, i.e., patient attended.

For the qualitative component, 11 healthcare providers were selected using purposive sampling based on cadre diversity, years of experience, and direct involvement in hypertension care, and recruitment continued until data saturation was reached.

### Barriers that influence healthcare provider adherence

Several barriers affecting healthcare provider adherence to the NSTGs in the management of hypertension emerged from the interviews. These barriers were grouped into four main themes: guideline-related, resource-related, provider-related, and patient-related barriers.

#### Guideline-related barriers.

Providers perceived the NSTGs as outdated noting that newer medications are not included and updates occur infrequently. As one provider explained, *“Some things in the STG are outdated. Many new drugs exist but aren’t in the STG. A patient comes with a drug, you check the STG, it’s not there, yet it’s effective”* (Medical Doctor). Participants suggested that guideline revisions should occur more frequently and be informed by locally generated evidence. One specialist noted, *“They should update the STG earlier. Waiting four or five years is too long… at least every two to three years”* (Specialist Physician).

Providers also highlighted limited guidance for managing hypertension with comorbidities, stating that some clinical scenarios are insufficiently addressed. *“The management is generalized… for hypertension and diabetes it’s explained, but for other categories the steps are not clear”* (Medical Doctor). Additionally, participants emphasized the need for greater involvement of frontline healthcare workers during guideline development to ensure recommendations are feasible in district hospital settings. *“STG development should involve users more… some things written in the STG, district hospitals can’t achieve due to limited resources”* (Medical Doctor).

Policy-related prescribing restrictions were also reported to limit adherence in some cases, particularly when medications initiated by specialists could not be continued by providers at lower levels. *“Some drugs require a physician to prescribe… a patient may come already started on medication by a specialist, but here I can’t prescribe it”* (Medical Doctor). Providers further suggested that regular supervision could help reinforce guideline use.

#### Resource barriers.

Limited availability of medications recommended in the NSTGs was frequently reported, particularly for hypertensive emergencies. One participant explained, *“IV medications, honestly, if you get them, it’s by luck. Hydralazine you might get by luck, labetalol there’s none… you have to mix and match”* (Assistant Medical Officer).

Shortages of diagnostic equipment and laboratory services also hindered guideline adherence. Providers described inadequate blood pressure monitoring equipment and limited access to investigations required for appropriate management. *“We have just one BP machine running around… tests for renal failure aren’t done yet because the lab isn’t fully operational”* (Medical Doctor).

In addition, specialized investigations such as echocardiography, ECG, and fundoscopy were often unavailable at district hospitals, requiring referrals to higher-level facilities. *“For ECHO and ECG tests we send patients to the regional hospital… our hospital still doesn’t have the capacity”* (Specialist Physician). Heavy patient loads and limited bed capacity further constrained the ability to manage patients according to guideline recommendations.

#### Provider-related barriers.

Participants reported limited formal training on the NSTGs, which affected familiarity with the guidelines. One provider noted, *“Two or three doctors were taken for training in Dodoma… but I’ve never seen if they came back with a guideline”* (Assistant Medical Officer). High staff turnover further reduced knowledge retention, as trained providers were often transferred to other facilities. *“Of those doctors who went for training, only one has remained… the others were transferred, so the knowledge is lost”* (Assistant Medical Officer).

Some providers also reported relying primarily on clinical experience rather than consulting the guidelines directly. As one participant stated, *“Blood pressure medications are well known; it’s almost routine… to say I refer to the guideline when seeing a patient—rarely”* (Medical Doctor).

#### Patient-related barriers.

Financial constraints among patients were commonly reported to affect adherence to recommended investigations and medications. *“Tests for patients with hypertension and other NCDs are very costly… the challenge comes when you need to move a patient to more advanced, expensive drugs”* (Medical Doctor).

Limited patient health literacy was also identified as a challenge, with some patients failing to fully understand their condition or the importance of treatment adherence. *“You may educate them and prescribe treatment, but on the next visit you find complications because they only bought a few of the medicines”* (Clinical Officer). Providers further emphasized the need for improved community awareness about hypertension and its risk factors. *“Many lack knowledge… even older patients don’t realize age itself is a risk for hypertension”* (Medical Doctor).

### Facilitators that enhance adherence

#### Supportive practices and resources.

Providers highlighted peer support, mentorship, and CME sessions as key facilitators.

Structured CME sessions and mentorship reinforced adherence. *“For training, we sometimes have doctors from programs like Mama Samia’s on-job training or from other facilities, which is good. We’ve learned a lot from Mama Samia’s program, where specialists come to mentor us. We ask questions, see where we’re going wrong, and improve… You can also learn on the job or visit other facilities to see how they treat patients and run their clinics”* (Medical Doctor).

Another provider added, *“Yes, we have CME every Tuesday. We have different departments, and the presentations vary. For example, OPD might present on hypertension or malaria on a given Tuesday. First, it helps you not forget things. You might forget something, but the NSTGs helps in treating the patient properly, especially with dosage—someone might forget, or in an emergency, you can refer to it, and the patient gets the right treatment”* (Assistant Medical Officer).

On-the-job training was also valued: *“Yes, I’ve had on job training. When you encounter a case and a senior doctor explains it to you, that’s on job training. If you see a similar patient the next day, you know where to start”* (Clinical Officer).

Digital access to the NSTGs in the management of Hypertension was noted as helpful: *“I have a hardcopy, I was given one…but the online one is available, you can open GOTHOMIS and on the side open the STG because it’s there, if you search for it on the computer, it comes up. The challenges I see: first, availability of resources. Hard copies are scarce, so soft copies are needed since they’re quicker to access, especially with many patients”* (Specialist Physician).

## Discussion

This study revealed that only 26.2% of patients with Hypertension at public district hospitals in Dar es Salaam received management that was completely adherent to the NSTGs consistent with evidence from Dodoma where 29.29% of patients received guideline-concordant management across all conditions [15]. This reflects systemic challenges across public health facilities in Tanzania that hinder consistent application of standardized protocols.

Comparable trends have been observed in other sub-Saharan African settings, including Ethiopia (19.5%), Ghana (30%), and Zimbabwe (35%) [13,16,17], these can be explained by inadequate provider training and resource limitations. Conversely, higher adherence in better-resourced tertiary hospitals, such as 67.1% in Nigeria, underscores the critical role of institutional support, continuous professional development, and resource availability in facilitating evidence-based practice [18].

Our findings indicate that patients attending follow-up visits were markedly more likely to receive guideline-concordant care, aligning with evidence from the United States and Indonesia that ongoing care enhances patients’ health literacy, thereby supporting consistent guideline application [19,20]. Regular clinic visits likely allow providers to build familiarity with patients’ treatment plans, facilitating adherence, whereas initial consultations may challenge guideline application due to incomplete patient histories. Similarly, the presence of comorbidities roughly doubled the likelihood of guideline-concordant management, consistent with a finding in South Africa for patients with Hypertension comorbid with Diabetes Mellitus [12].

Furthermore, adherence was higher in the management of patients with Grade 1 and Grade 2 hypertension compared with controlled patients, although adherence for Grade 3 hypertension was not statistically significant, likely due to the smaller number of patients in this category. This pattern suggests that providers may be more likely to follow guideline recommendations when managing patients with evident hypertension severity, while lower adherence in controlled cases may reflect therapeutic inertia, where treatment adjustments are less aggressively pursued despite ongoing guideline recommendations.

In this study healthcare provider adherence was not associated with patient age, sex, nor mode of payment. This contrasts with a study in the United States which suggested that demographic factors like age and gender may necessitate deviations from guidelines due to individualized needs [21].

Specialists showed a sevenfold higher prevalence of complete adherence compared to clinical officers, consistent with studies in Nigeria [18] and Malaysia [22] linking provider knowledge to guideline adherence [21,23]. Their advanced training and exposure to evidence-based practices could enable more accurate application of the NSTGs, whereas clinical officers, who may have less formal training, might rely more on informal mentorship, as suggested by the qualitative data. The wide confidence interval suggests limited precision, possibly due to small subgroup sizes, highlighting the need for larger studies to confirm this effect.

Providers with five or more years of experience showed 3–4-fold higher adherence compared to those with less than five years, consistent with a study in Sudan linking adherence to clinical experience [24], but contrasting a study in United States where less experienced providers relied heavily on guidelines [21]. In Dar es Salaam, experienced providers likely develop practical skills enabling better NSTG application, while newer providers face challenges due to limited training.

Guideline-related factors emerged as significant barriers. Providers reported that the current edition of NSTGs is outdated, lacking updated drug regimens and protocols, and provided limited guidance for managing comorbidities, compelling reliance on clinical judgment or external references. The limited involvement of frontline providers in guideline development further reduced relevance to local practice, echoing findings from Zimbabwe that inclusive guideline development enhances adherence [25]. Resource constraints, including persistent drug shortages, malfunctioning or unreliable blood pressure monitors, and frequent stock-outs of essential supplies, substantially hindered consistent application of the NSTGs. These challenges were compounded by high patient volumes and heavy workloads, which limited the time healthcare providers could dedicate to thorough clinical assessment and documentation. Inadequate supervision and irregular clinical audits further weakened accountability and opportunities for feedback, leading to variability in practice and reduced motivation to adhere strictly to guidelines.

The systemic and structural barriers identified in this study mirror broader evidence from sub-Saharan Africa, where the absence of supportive health system environments, including adequate staffing, continuous supervision, and reliable resources, has been shown to undermine adherence to standard treatment guidelines. Key challenges include inadequate staffing, insufficient supervision, and unreliable availability of essential resources, which collectively reduce the quality of hypertension care. These obstacles contribute to poor detection, treatment, and control rates, while reliance on data from non-native populations may further limit treatment effectiveness. [23,24,26–29].

Socioeconomic factors, including high out-of-pocket costs and low health literacy, also contributed to deviations from recommended care, underscoring the influence of patient context on provider adherence. Similar observations were reported in Zimbabwe, where financial barriers and limited awareness hindered blood pressure control [25], and in Nigeria, where inadequate insurance coverage and poor understanding of hypertension reduced quality of care [23]. Studies from Ghana and Tanzania further highlight that limited patient knowledge and poor facility preparedness continue to impede effective hypertension management [26,30].

Facilitators of adherence included peer support and mentorship, access to digital NSTGs, and CME sessions, which enhanced guideline use even in resource-limited settings. Providers highlighted that peer consultation and informal mentorship helped in complex cases, while digital copies allowed rapid reference when hard copies were unavailable. This was similarly observed in Saudi Arabia and Malaysia [31,32].

Overall, these findings indicate that adherence to hypertension management guidelines in Dar es Salaam is shaped by patient complexity, provider expertise, guideline relevance, and systemic factors, including resources, supervision, and workload.

### Strengths and limitations of the study

This study has several strengths. Its mixed-methods design, combining retrospective analysis of 397 patient files with 11 in-depth interviews, provides a comprehensive assessment of guideline adherence across five public district hospitals spanning all districts of Dar es Salaam. Data collection used a structured checklist based on the 6th edition NSTGs cross-verified with GOTHOMIS and manual registers, enhancing reliability. Integration of quantitative and qualitative findings further strengthens the credibility and depth of the conclusions.

However, several limitations should be noted. The cross-sectional design prevents causal inference, and focus on urban hospitals limits generalizability to rural settings. Retrospective file review introduced potential inaccuracies, with 16.2% of files excluded due to missing information. Non-pharmacological interventions were frequently under-documented, limiting the scope of adherence assessment. The composite adherence measure used a 65% threshold for complete adherence, adopted from prior regional studies using comparable multi-domain checklists, which may have led to overestimation of complete adherence.

Resource barriers were captured qualitatively through provider interviews rather than a structured facility readiness assessment, which may not fully quantify objective facility-level deficiencies. The qualitative component did not differentiate responses by provider cadre, patient perspectives were not included, and small subgroup sizes resulted in wide confidence intervals for some estimates.

## Conclusion and recommendations

Adherence to the NSTGs for hypertension management in public district hospitals in Tanzania remains suboptimal, increasing the risk of preventable complications. Several key barriers and facilitators influencing adherence were identified, spanning guideline-related, resource-related, provider-related, and patient-related factors.

At the policy level, the Ministry of Health should regularly update the NSTGs every two to three years to reflect current evidence and local contexts, with active involvement of frontline providers in guideline development, dissemination, and sensitisation. Incorporating adherence indicators into supportive supervision frameworks and district performance assessments would strengthen accountability. In the longer term, expanding health insurance coverage and introducing subsidy mechanisms for essential diagnostics, medicines, and follow-up services could reduce out-of-pocket costs for patients.

At the facility level, investments in training, infrastructure, supply chain management, and routine clinical audits should be prioritised alongside peer mentorship programmes. Further research should incorporate structured facility readiness assessments tools to objectively quantify resource availability alongside provider-reported experiences, assess adherence in rural and diverse settings, include patient perspectives, and link provider practices to clinical outcomes. Coordinated policy, facility-level, and research efforts are essential to improve guideline adherence and hypertension outcomes nationwide.
