# Supplementary material for: Adherence to National Standard Treatment Guidelines in the management of hypertension and associated factors among healthcare providers at public district hospitals in Dar es Salaam, Tanzania
Source: PLOS Glob Public Health. 2026 Jul 9;6(7):e0005818. doi: 10.1371/journal.pgph.0005818 (PMC13349132; doi:10.1371/journal.pgph.0005818)
Supplement: Attachment — Submitted filename: Response to reviewers.docx [file pgph.0005818.s003.docx]

Dr. Gasper Singfrid Mung’ong’o,
P.O. Box 13363
Dar es Salaam, Tanzania
+255 685 661 790
gascav1994@gmail.com

PLOS Global Public Health
PLOS
1875 Mission Street
Suite 103 #188
San Francisco, CA 94103
United States

Dear Reviewers,

**SUBJECT: RESPONSE TO REVIEWERS COMMENTS ON MANUSCRIPT**

We sincerely thank you for your careful review of our manuscript entitled *“Adherence to National Standard Treatment Guidelines in the Management of Hypertension and Associated Factors Among Healthcare Providers at Public District Hospitals in Dar es Salaam, Tanzania.”*

We have carefully considered each of your comments and suggestions. Our detailed, point-by-point responses are provided in the response matrix below. Where applicable, we have revised the manuscript to address your concerns, clarify methodology, and improve readability.

We greatly appreciate your constructive feedback, which has helped strengthen our manuscript.

Sincerely,

Dr. Gasper Singfrid Mung’ong’o,

MD, MSc PMMEH
On behalf of all authors

**RESPONSE MATRIX**

| **Section** | **Comments from Reviewer** | **Author response** | **Line number where the changes have been made** |
| --- | --- | --- | --- |
| **General – throughout manuscript** | For all the manuscript, correct “hypertensive patient” to “people with hypertension” | We have replaced all instances of “hypertensive patient” with “patients with Hypertension”. | 94, 117, 128, 131, 138, 175, 194, 238, 267, 66, 99, 117 |
| **Abstract – Materials and Methods** | Quantitative data from 397 patient files : It is already a result. Insert it among results. | We have moved the number (397 patient files) from Methods to Results section of the abstract. | 39 |
| **Abstract – Materials and Methods** | Why Poisson regression and not a linear one? | We used it because our outcome was categorical- binary variable and it had prevalence >10%. Here odds ratios can overestimate effects, so Modified Poisson allows direct estimation of prevalence ratios for a more accurate and interpretable association. | 36-37 |
| **Abstract – Results** | there is no characteristics of people living with HTN | We have added key patient characteristics including median age, sex, BMI category and presence of comorbidities. | 39-40 |
| **Abstract – Results** | add p-value on the regression results: aPR = 7.00; 95% CI: 3.08–15.92, p= | We have added the corresponding p-values on the regression results for all associated factors. | 45 |
| **Introduction** | Line 74: … propose you to say that there is a discrepancy between urban and rural prevalence of HTN. | We have substantially strengthened this section by not only highlighting the disparity but also elaborating on the multifactorial drivers that underpin it, drawing on established epidemiological evidence. | 76-82 |
| **Introduction** | Line 76–77: limited access to care is more global | We have expanded to demonstrate how globally there are issues with inadequate screening, and inadequate blood pressure control among patients on treatment. | 83-88 |
| **Introduction** | Line 91: specify which guidelines please | We explicitly stated “National Standard Treatment Guidelines (NSTGs), 6th edition (2021)”. | 99 |
| **Materials and Methods** | Line 97: No need to precise that is analytical… either you say that this study had 2 components… | We removed the word “analytical” | 107 |
| **Materials and Methods** | You stayed mixed methods, precise which one | We clarified it as: convergent parallel mixed-methods. | 107-108 |
| **Materials and Methods** | Line 110: outpatients departments or consultations? | We clarified it by stating that patients who attended their outpatient clinics for Hypertension. | 120-121 |
| **Materials and Methods** | Line 111: all cadres ??? What do you want to say ? any type ??? | Yes, here we meant all healthcare providers who prescribe for and manage patients with Hypertension. | 120-122 |
| **Materials and Methods – Inclusion criteria** | Line 118: rephrase both provider and patient inclusion criteria (grammar & clarity) | We rewrote the inclusion criteria for better grammar and clarity | 127-131 |
| **Materials and Methods – Exclusion criteria** | Line 125: did you include Pregnant women??? or Retraction from the study??? | Pregnant women were not systematically excluded if they met other criteria; however, pregnancy with hypertension cases were captured as comorbidity. No participants withdrew. | 127-131 |
| **Materials and Methods – Sample size** | Line 130: You did have any minimum sample size??? | We added explanation All eligible records from the 12-month period were included (census approach) to maximize power and minimize selection bias; no a priori minimum sample size was calculated. | 138-140 |
| **Materials and Methods – Qualitative** | Line 134: I would have prefered that you have a structured questionnaire. qualitative interview are biased | We acknowledged limitation of semi-structured interviews (Hawthorne effect) and explained use of topic guide + triangulation + member checking to enhance trustworthiness. | 196-197 |
| **Materials and Methods** | Line 165: what is ODK | We added “Open Data Kit (ODK) – a free, open-source mobile data collection platform”. | 174 |
| **Materials and Methods** | Line 208: what is ATLAS.ti ? | We added: “ATLAS.ti (version 22) – a qualitative data analysis software”. | 219-220 |
| **Results – Figure 1** | What were the ineligibility criteria? | The ineligibility criteria were the exclusion criteria outlined in the materials and methods section. | 132-136 |
| **Results – Qualitative** | Line 262: You choose some healthcare providers. Why and how did you choose them? | We clarified in text: Purposive sampling based on cadre diversity, years of experience, and direct involvement in hypertension care; continued until data saturation. | 264-266 |
| **Results – Tables 2 & 3** | Line 272: P-value ??? You gave the p-value for follow-up visits, present similarly grade 1, 2 and 3 hypertension | We added the missing p value | 279 |
| **Results – Barriers** | Presentation of barriers is too long (almost 5 pages). Please try to reduce it | We condensed the barriers section to almost 50% by grouping similar quotes, reducing repetitions, and focusing on most salient themes and representative quotations. | 14-75 |
| **Discussion** | Line 129: Did these studies prove what are you stating? | We have revised to state that these studies reported similar low adherence levels and … potentially explained by…” (rather than stating they “proved” the barriers). | 104-106 |
| **Discussion** | Line 143: was also higher in management of patients with Grade 1 and 2. Is it not indicative of therapeutic inertia? | Yes, this is indicative of therapeutic inertia. We have added a sentence on how providers may be reluctant to take more action when managing controlled hypertension | 122-124 |
| **Discussion** | Line 153: Use the conditional tense. Be less assertive. | We have revised the tense to be less assertive | 131-134 |
| **Discussion / References** | Line 177: Please cite Hypertension in Sub-Saharan Africa: Burden, Barriers and Priorities… (Doi: 10.1161/CIRCRESAHA.124.323889) | We have incorporated this review into the section | 155-162 and reference number 40. (line 360-362) |
