# Supplementary material for: Adherence to National Standard Treatment Guidelines in the management of hypertension and associated factors among healthcare providers at public district hospitals in Dar es Salaam, Tanzania
Source: PLOS Glob Public Health. 2026 Jul 9;6(7):e0005818. doi: 10.1371/journal.pgph.0005818 (PMC13349132; doi:10.1371/journal.pgph.0005818)
Supplement: Attachment — Submitted filename: Response_to_reviewers_auresp_2.docx [file pgph.0005818.s004.docx]

Dr. Gasper Singfrid Mung’ong’o,
P.O. Box 13363
Dar es Salaam, Tanzania
+255 685 661 790
gascav1994@gmail.com

PLOS Global Public Health
PLOS
1875 Mission Street
Suite 103 #188
San Francisco, CA 94103
United States

Dear Reviewers,

**SUBJECT: RESPONSE TO SECOND REVIEWERS COMMENTS ON MANUSCRIPT**

We sincerely thank you for your careful review of our manuscript entitled *“Adherence to National Standard Treatment Guidelines in the Management of Hypertension and Associated Factors Among Healthcare Providers at Public District Hospitals in Dar es Salaam, Tanzania.”*

We have carefully considered each of your comments and suggestions. Our detailed, point-by-point responses are provided in the response matrix below. Where applicable, we have revised the manuscript to address your concerns, clarify methodology, and improve readability.

We greatly appreciate your constructive feedback, which has helped strengthen our manuscript.

Sincerely,

Dr. Gasper Singfrid Mung’ong’o,

MD, MSc PMMEH
On behalf of all authors

**RESPONSE MATRIX**

| **Section** | **Comments from Reviewer** | **Author response** | **Line number where the changes have been made** |
| --- | --- | --- | --- |
| **Introduction** | The introduction is too long and should focus more on the problem of adherence to standard treatment guidelines. | We agree with this comment.  The introduction had too much background information on epidemiology, which took attention away from the main issue. We shortened the parts about the global burden, NCDs in Tanzania, and urbanization.  We now clearly state the key gap earlier. The revised introduction now explains the research problem better and why the study is needed. | 55-79 |
| **Materials and Methods- Data analysis** | Data analysis (P9, line 212)- The authors justify the use of the adherence cutoffs (e.g. 65% is good) based on prior studies that used the same cutoff.  However this is a very low cutoff and there are data to show that this level of adherence would negatively affect patient outcomes.  A better method should be considered or stronger justification for using these cutoffs. | We thank the reviewer for this observation.  The available clinical evidence shows that patient medication adherence below 80% leads to poorer outcomes.  We would like to clarify:  1. First, in this study adherence is a composite variable that also includes: screening for comorbidities and monitoring for complications in addition to medications adherence. These three components have been adapted from standard treatment guidelines.  2. Second, our study focuses on the provider’s perspective, i.e adherence to the guidelines which may differ from patient adherence to prescribed medication.  The ≥65% cutoff was therefore applied as an **operational classification threshold** to distinguish relatively higher versus lower levels of guideline concordance, consistent with prior studies employing similar composite approaches in comparable settings. This facilitates contextual interpretation and comparability across studies.  Nonetheless, we have added this as a study limitation, that the threshold is low and recommended for future research to standardize the cut offs especially when investigating provider related adherence to guidelines. | 189-192, 191-193 |
| **Results – Quantiative** | Comment 3a: Line 279 — avoid causal language when describing associations. | We agree. The sentence has been revised to: "Presence of comorbidities was significantly associated with a higher prevalence of guideline-concordant management (aPR = 2.35; 95% CI: 1.25–4.41; p = 0.008)." The remainder of the Results section has been reviewed and causal language removed throughout. | 257-258 |
| **Results – Qualitative** | Is it possible to quantify the resource barriers e.g. by systematically reporting level of availability of essential drugs and equipment in the participating hospitals? There are tools such as the WHO SARA tool that can be used to do this | Thank you for this comment.  We acknowledge that a structured facility assessment tool like the WHO SARA, is very useful for measuring the availability of supplies quantitatively.  In our study, we wanted to understand healthcare providers’ unique experienced barriers and facilitators as part of a process evaluation study examining fidelity and context of the NSTGs implementation. We chose qualitative approach because it is well suited to capture these experiences.  We have added that not having a formal facility readiness assessment as a limitation. We have also added a recommendation for future studies to combine qualitative exploration with quantitative tools. This would provide objective facility-level data alongside what providers report from their experience. | 211-213, 193-195 |
|  |  |  |  |
|  |  |  |  |
